# Development and psychometric evaluation of an instrument for medical students’ self-assessment of professionalism: Reliability, content, and construct validity of the MediProf questionnaire

**DOI:** 10.3205/zma001834

**Published:** 2026-03-23

**Authors:** Larissa Fey, Stefanos A. Tsikas, Sarah Meissner, Anja Hesse, Kambiz Afshar

**Affiliations:** 1 Hannover Medical School, Institute for General Practice and Palliative Care, Hannover, Germany; 2 Hannover Medical School, Dean of Studies Office, Academic Controlling, Hannover, Germany

**Keywords:** Professional Identity Formation, professionalism, professional development, medical education, physician identity, physician roles, competencies, questionnaire

## Abstract

**Objective:**

The aim of this study was to develop a self-assessment instrument for measuring medical professionalism among medical students and to conduct an initial evaluation of its psychometric properties.

**Methods:**

The *MediProf *questionnaire was developed in an iterative process based on the *Professionalism Scale Germany (Pro-D)*. It comprises a total of 64 items, of which 61 are rated on a four-point Likert scale (1=strongly disagree; 4=strongly agree) and cover four dimensions of medical professionalism: towards 1) oneself, 2) patients, 3) other healthcare professions, and 4) society. Three items assess the relevance and the extent to which the topic is addressed at the respondents’ own institution. Comprehensibility and feasibility were evaluated in two pretests. In 2024, the questionnaire was administered online to 224 medical students. Statistical analyses were conducted using SPSS and Stata. In addition to calculating scale scores per dimension, item discrimination, and item difficulty, we assessed internal consistency using Cronbach’s alpha (α) and conducted a confirmatory factor analysis. Multiple linear regression analyses were used to examine the influence of sociodemographic factors on self-assessed professionalism.

**Results:**

Analysis of the complete questionnaire data from 155 students demonstrated good internal consistency for the *MediProf *questionnaire across all 61 items (α=0.90), with good to acceptable values for the individual dimensions: 1) α=0.84; 2) α=0.75; 3) α=0.77; and 4) α=0.62. Most items showed good discrimination. The factor analysis indicated an overall relationship between the items and the four dimensions, with high interdependencies and weak factor loadings. Students rated their professionalism overall as high (mean=3.3; SD=0.2). Regression analyses revealed no significant influence of sociodemographic variables on self-assessed professionalism. The majority of participants considered the topic very important (94.5%) and called for stronger curricular integration (79.5%).

**Conclusions:**

The newly developed *MediProf* questionnaire provides, for the first time, a German-language self-assessment instrument with good psychometric properties for measuring medical professionalism among medical students. The participants’ expressed desire for stronger curricular integration highlights the importance of early and continuous promotion of professionalism throughout medical education.

## Zusammenfassung

**Ziel:**

Ziel dieser Arbeit war die Entwicklung eines Selbsteinschätzungsinstruments zur Erfassung ärztlicher Professionalität im Medizinstudium und die erste Überprüfung seiner psychometrischen Eigenschaften.

**Methodik:**

Der Fragebogen *MediProf* wurde basierend auf der *Professionalitätsskala Deutschland* in einem iterativen Prozess entwickelt. Er umfasst insgesamt 64 Items, von denen 61 Items auf einer vierstufigen Likert-Skala (1=stimme überhaupt nicht zu; 4=stimme voll und ganz zu) bewertet werden, und vier Dimensionen ärztlicher Professionalität abdecken: gegenüber 1) sich selbst, 2) Patient*innen, 3) anderen Berufsgruppen im Gesundheitssystem und 4) der Gesellschaft. Drei Items erfragen die Relevanz und den Grad der Abdeckung des Themas am eigenen Standort. Verständlichkeit und Praktikabilität wurden in zwei Pretests überprüft. Der Fragebogen wurde 2024 in einer Online-Erhebung mit 224 Medizinstudierenden erprobt. Die statistische Analyse erfolgte mit SPSS und Stata. Neben der Berechnung von Skalenwerten pro Dimension, Trennschärfe und Itemschwierigkeit wurde die interne Konsistenz durch Cronbachs Alpha (α) überprüft und eine konfirmatorische Faktorenanalyse durchgeführt. Multiple lineare Regressionsanalysen untersuchten den Einfluss soziodemografischer Faktoren auf die selbsteingeschätzte Professionalität.

**Ergebnisse:**

Die Auswertung der vollständigen Fragebogendaten von 155 Studierenden zeigt eine gute interne Konsistenz des *MediProf*-Fragebogens über alle 61 Items (α=0,9) sowie gute bis akzeptable Werte für die einzelnen Dimensionen: 1) α=0,84; 2) α=0,75; 3) α=0,77; und 4) α=0,62. Die meisten Items sind trennscharf. Die Faktorenanalyse zeigt eine generell vorhandene Beziehung zwischen den Items und den vier Dimensionen bei hohen Interdependenzen und schwachen Faktorladungen. Die Studierenden schätzten ihre Professionalität insgesamt hoch ein (MW=3,3; SD=0,2). Die Regressionsanalyse zeigte keinen signifikanten Einfluss soziodemografischer Variablen auf die Selbsteinschätzung der Professionalität. Die Mehrheit der Teilnehmenden hielt das Thema für sehr wichtig (94,5 %) und forderte eine stärkere curriculare Verankerung (79,5 %).

**Schlussfolgerungen:**

Mit dem neu entwickelten Fragebogen *MediProf *steht erstmalig ein deutschsprachiges Selbsteinschätzungsinstrument mit guten psychometrischen Eigenschaften für die Erfassung ärztlicher Professionalität von Medizinstudierenden zur Verfügung. Der von den teilnehmenden Medizinstudierenden geäußerte Wunsch nach stärkerer curricularer Integration unterstreicht die Bedeutung einer frühzeitigen und kontinuierlichen Förderung der Professionalisierung im Studium.

## 1. Introduction

The development of medical professionalism is a central goal of medical education, as it encompasses not only clinical knowledge but also the ethical and social competencies necessary for future practice [1]. In addition to technical expertise, aspects such as responsibility, empathy, and the capacity for reflection play a crucial role [2]. The process of Professional Identity Formation (PIF), which describes the development of a physician’s identity throughout training, is of fundamental importance, as it has a long-term influence on students’ professional attitudes and behaviour [3], [4]. Medical education is not only about acquiring knowledge but also about establishing oneself as a reflective and ethically responsible healthcare professional [5]. PIF involves a variety of factors, including social learning through interactions with peers and patients, as well as individual engagement with personal professional values [6]. This process is significantly shaped during medical school, and targeted support for PIF has long-term positive effects on professional satisfaction and the quality of patient care [7], [8]. Particularly in collectivist and hierarchically structured cultures, the development of a professional identity can be strongly influenced by these factors [3].

### 1.1. Theoretical framework and concept of professionalism

A central theoretical model for the development of professional identity is provided by Chandran et al. [9]. According to this approach, professional identity develops through the dynamic interaction of individual, relational, and collective identities within a so-called “community of practice”. Individual identity encompasses personal values, motivation, and the capacity for reflection, while relational identity is shaped through interactions with peers, mentors, and patients. Collective identity emerges within the medical community, where shared values and norms are conveyed and internalised [10]. This model, along with other studies, highlights that Professional Identity Formation (PIF) is not an isolated process but occurs within a social and cultural context [11]. Accordingly, the identity development of medical students is influenced not only by formal curricula but also by informal learning processes, such as those occurring in clinical settings and interactions with experienced professionals. Conscious reflection on these influences can help foster a stable and authentic professional identity that integrates both individual and collective values. Key theoretical foundations for PIF also include the CanMEDS framework [12] and the model by van Mook et al. [13]. These models define physician competencies and roles and address the development of professional identity across different stages of training. The CanMEDS competency framework provides a detailed structure for various dimensions of medical professionalism, such as medical expertise, communication, teamwork, and leadership [12], and serves as a basis for both postgraduate training and undergraduate medical curricula. Another important concept for the development of professionalism in medical education is proto-professionalism, as described by Hilton and Slotnick [14]. They emphasise that medical students develop an early form of professionalism during the initial stages of training, which lays the foundation for the later emergence of a fully developed professional identity. This formative phase is critical for the subsequent integration of professional values. A key element of PIF is the continuous reflection on one’s professional actions [4], enabling students to critically examine their developing professional identity. Combined with social learning processes, professionalism is thus acquired not only cognitively but also through experiences in real clinical contexts.

### 1.2. Problem statement and research gap

In Germany, there are occasional university-based initiatives addressing professionalism in medical education, such as the “LET ME” programme in Munich [15] or “LongProf” in Jena [16]. Existing teaching methods and curricula aim to promote engagement with the topic of professionalism; however, a structured, validated feedback mechanism that enables students to actively shape their professional identity is lacking. Despite the recognized value of Professional Identity Formation (PIF) in medical education, there are currently few standardized approaches that systematically allow medical students to reflect on and measure their own development in terms of professionalism [5], [7]. The existing literature discusses various theoretical models of PIF, yet practical, validated instruments to assess this developmental process within the specific context of medical education are scarce [17], [18]. For postgraduate medical training, the *Professionalism Scale Germany (Pro-D)* provides a German-language instrument for evaluating professional identity development [19]. Initial approaches for the student context also exist, such as the Professional Self Identity Questionnaire (PSIQ) by Vivekananda-Schmidt et al., which has been applied to students across various healthcare professions [20], [21]. Overall, however, the number of established, theory-driven instruments for measuring PIF in the German-speaking context remains limited. Against this background, adapting the Pro-D – originally developed for postgraduate training – appears appropriate, as this instrument captures reflective engagement with professional attitudes across four dimensions, making it structurally suitable for use in medical education.

### 1.3. Study objective and research questions

The primary aim of this study was to develop a self-assessment instrument for measuring medical professionalism in undergraduate medical education. The questionnaire is intended to help medical students reflect on and actively foster their own professional identity and development. In addition, the psychometric properties of the instrument were examined to provide initial evidence of its internal consistency (reliability) as well as content and construct validity. As processes of professionalisation and professional identity formation can be influenced by biographical and contextual factors [6], [22], the study also aimed to investigate the impact of sociodemographic characteristics on self-assessed professionalism. Previous research indicates, for example, differences in professional identification and role adoption depending on the year of study [23], with stronger professional development expected as clinical exposure increases. Age may be associated with greater biographical maturity and reflective capacity [24], which can positively influence the development of professional attitudes. Gender is also discussed in the context of professional socialisation, particularly regarding communication styles, role expectations, and social attributions [25]. Prior experience – such as completed vocational training or previous exposure to the medical field – can be considered a form of “informal professionalisation” [26], providing students with early insights into medical routines and professional expectations before entering medical school.

In this context, the following research questions were addressed:

1. What are the psychometric properties of the developed questionnaire for assessing medical professionalism in undergraduate medical education, and what insights can be derived regarding its internal consistency as well as content and construct validity?
2. How do medical students rate their own professionalism across different dimensions of medical practice?
3. To what extent do factors such as gender, age, completed vocational training, prior experience in the medical field, and year of study influence self-assessed professionalism?

In addition, the following questions were considered to assess the topic of professionalisation in medical education at the institutional level:

1. How relevant do medical students consider the topic of medical professionalism for their education?
2. To what extent do medical students desire curricular integration of medical professionalism at their own institution?

## 2. Methods

### 2.1. Questionnaire development

A German-language questionnaire for assessing medical students’ professionalism does not yet exist. Given the central role of self-reflection and reflective capacity in the process of Professional Identity Formation (PIF) and medical professionalisation [2], [4], [27], the *“Assessment of Medical Professionalism in Undergraduate Medical Education” (MediProf)* was developed as a self-assessment instrument. The questionnaire was based on the *“Professionalism Scale Germany” (Pro-D)* [19], which itself was derived from the Dutch* “The Nijmegen Professionalism Scale”* [28]. This scale was chosen due to its comprehensive theoretical foundation and its structural similarity to PIF concepts, particularly the model described by Vignoles et al. [10] and represented by Chandran et al. [9], which considers individual, relational, and collective identities.

The adaptation for medical students was carried out in several steps. First, items that were not applicable (e.g., “I can justify the indication for a home visit”) were identified. The remaining items were then adapted to the context and target group. This iterative process was conducted within an interdisciplinary team (general medicine, medical education, psychology). Items were evaluated based on the following criteria:

1. relevance for medical students,
2. clarity, and
3. theoretical foundation within the context of PIF.

The development of new items was guided by overarching competencies from the National Competency-Based Catalogue of Learning Objectives for Medicine (NKLM) ([https://nklm.de/zend/menu]; last accessed: 20 June 2025), findings from a Canadian interview study with medical students during their clinical electives [29], and experiences from a pilot project on longitudinal professionalisation in medical education at the University of Jena [16].

The *MediProf *questionnaire assesses four dimensions of medical professionalism (see table 1 (Tab. 1)), which are based on the levels of individual, relational, and collective identity [9], [10].

Table 1 (Tab. 1) presents the four dimensions of professionalism from the *MediProf* questionnaire, including example items and a comparison with the *Pro-D* questionnaire

The *MediProf* questionnaire comprises 64 items: 61 items for self-assessment of professionalism and 3 items assessing the integration of professionalisation in medical education at the students’ own institution. Table 2 (Tab. 2) provides an overview of the instrument’s composition.

The complete *MediProf* questionnaire can be found in attachment 1 . Attachment 2 provides an overview of all *MediProf* items alongside the corresponding items from the Pro-D, including details of the adaptation process (see attachment 2 , tables A2.1 and A2.2).

### 2.2. Pretest

Comprehensibility, in terms of content validity, and the practicability of the questionnaire were evaluated in two pretests. In the first pretest, six final-year medical students (Practical Year, PJ) and three instructors participated and provided written and oral feedback as needed. Linguistic ambiguities were identified and corresponding adjustments made. For example, the term “Verabredungen” in item 1.9 was discussed within the interdisciplinary team following participant feedback and changed to “Anregungen”. Further adjustments addressed consistent gender-sensitive language. The average completion time was 9 minutes and 23 seconds. In the second pretest, five students from years 2-5 completed the revised questionnaire as part of an elective on professionalisation. As no further issues arose, the questionnaire remained unchanged.

### 2.3. Pilot testing

#### 2.3.1. Recruitment and data collection

**Figure 1:**
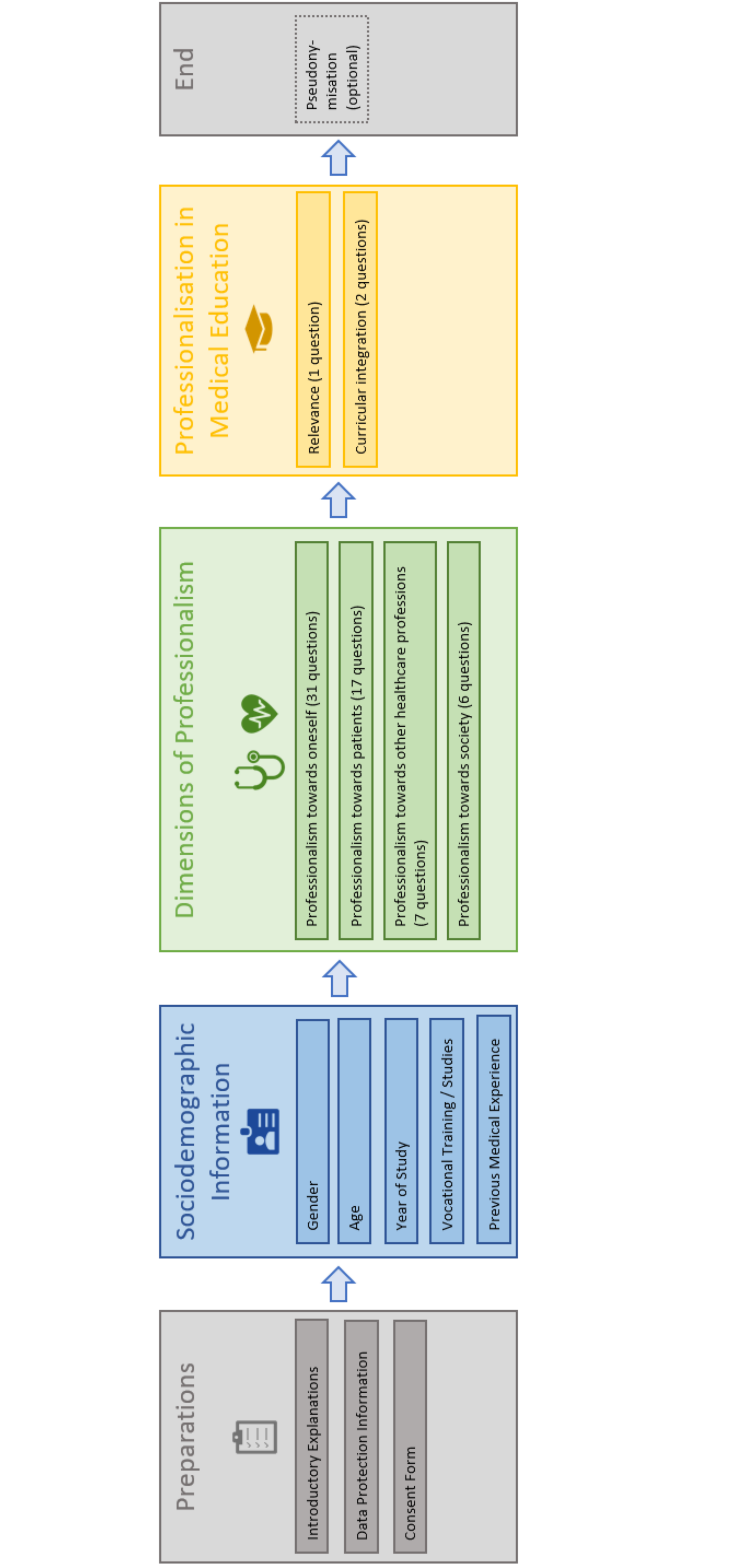
Online survey procedure

In April 2024, all enrolled medical students at Hannover Medical School (MHH) in years 1-6 (N=1,866) were invited via email through the dean’s office, evaluation & capacity department, with a survey link to participate. Participation was voluntary and conducted online without compensation using SoSci Survey [30]. Reminders were sent after four and seven weeks. In addition, posters on campus, AStA mailing lists, and the digital learning platform ILIAS were used for recruitment. The survey procedure is illustrated in figure 1 (Fig. 1).

#### 2.3.2. Sample

Of the 1,866 invited medical students, 224 (12%) participated in the survey. For the subsequent analyses, data from N=155 students were used, who had completed all items across the four dimensions in addition to providing sociodemographic information. In this sample, the proportion of missing item responses (i.e., “missing values”) per dimension was below 10% (dimension 1: 1.3%; dimension 2: 4.7%; dimension 3: 4.9%; dimension 4: 8.6%). The mean age of participants was 25.39 years (SD=5.83). Table 3 (Tab. 3) presents the participants’ sociodemographic characteristics.

### 2.4 Data analysis

Statistical analyses of the survey data were conducted using Microsoft Excel (Version 2016), SPSS (Version 29.0.1.0), and Stata (Version 14). The reliability of the *MediProf *questionnaire was assessed using several measures. To evaluate its psychometric properties, Cronbach’s alpha was calculated for the four dimensions and the overall scale. Defined on a scale from 0 to 1, higher alpha values indicate stronger inter-item correlations. Values above 0.6 indicate satisfactory internal consistency, while values above 0.8 indicate high internal consistency [31]. Item discrimination was also calculated to examine the extent to which each item correlates with its respective total scale (excluding the item itself). High discrimination indicates that an item effectively captures and differentiates the underlying dimension. Values above 0.4 are considered discriminating, whereas values below 0.2 suggest very low discriminatory power and may warrant item revision or removal [32], [33]. Finally, item difficulty was assessed to evaluate the level of agreement in responses. Balanced distributions (values around 0.5) are considered optimal, while very high (>0.8) or very low (<0.2) values indicate limited variation and potential ceiling or floor effects, which could affect reliability [31]. Detailed results for item-level indicators are presented in attachment 3 .

In addition to content validity, which was ensured through pretests and review by experts in medical education and clinical practice, construct validity was examined using confirmatory factor analysis (CFA). In the CFA, a structural equation model (SEM) was used to assess the extent to which the four predefined dimensions are operationalised by their assigned items (see table 2 (Tab. 2)). Full-Information Maximum-Likelihood (FIML) estimation was applied in the SEM to handle missing item responses. SEM output tables are provided in attachment 3 , and fit indices are presented in section 4.2. The CFA, combined with psychometric parameters, provides insights into which aspects of professionalisation in medical students differ from those in postgraduate trainees [19] and identifies items that could be revised or removed due to redundancy or low factor loadings in future instrument development. For inferential analyses, descriptive statistics (mean, median, and standard deviation) were first calculated for each dimension and the overall scale to examine the data distribution. Responses of “cannot assess” were treated as missing values for the respective items. To investigate the influence of sociodemographic factors (age, gender, completed vocational training, prior medical experience, and year of study) on professionalism, multiple linear regression analyses were conducted using the mean professionalism score per dimension as the outcome variable. The first year of study was used as the reference category, as students in this year are generally expected to exhibit the lowest level of professionalisation. Differences in professionalism among higher study years were therefore measured relative to first-year students. Wald tests were additionally performed to determine whether the indicators for study year were jointly significant, i.e., whether there is an overall association between study progression and professionalisation. A significance level of α=0.05 was applied for all sample tests and regression analyses.

## 3. Results

The questionnaire data from N=155 students constitute the study population and serve as the basis for data analysis.

### 3.1. Psychometric properties of the MediProf questionnaire (research question 1)

Cronbach’s alpha indicated acceptable to good internal consistency across all dimensions. Dimension 1 showed the highest consistency (α=0.838), while Dimension 2 had α=0.747. As expected, alpha tends to increase with the number of items [31]. Accordingly, Dimension 3 (seven items) demonstrated good internal consistency with α=0.771. Only Dimension 4 (six items) showed a lower but still acceptable alpha of 0.615. For the overall *MediProf* questionnaire (61 items), α=0.90. Attachment 3 demonstrates that removing individual items has minimal impact on internal consistency. The majority of items within each dimension can be considered discriminating [31]; the item-total correlations in tables A3.1-A3.4, (see attachment 3 ) are at least >0.3 and often >0.4. A few items exhibited low discrimination, for example, “I can clearly and distinctly express my own opinion” (see attachment 3 , table A3.1; SE02_18) and “I can maintain emotional boundaries from patients’ emotions” (see attachment 3 , table A3.2; PA01_15). These items may warrant revision or removal. As shown in table 4, self-assessed professionalism was generally high with little variation around the mean, which is also reflected in high agreement rates (“item difficulty”) for some items (see attachment 3). In dimension 1, approximately 40% of items reached the threshold of 0.8 (dimension 2: 53%, dimension 3: 57%, dimension 4: 33%). Items such as “After an unpleasant conversation, I quickly regain composure” (see attachment 3 , table A3.1; SE02_16) effectively differentiate between respondents with lower or higher self-assessed professionalism, for example, regarding professionalism towards oneself. All psychometric parameters were also calculated using the maximum number of observations per item, which showed no substantial differences compared to the values reported in attachment 3 .

### 3.2 Confirmatory factor analysis (research question 1)

In the CFA, considering all 61 items (see table A3.5 in attachment 3 ), all items loaded significantly on their respective latent dimensions, indicating an overall relationship between the items and the proposed dimensions. However, the magnitude of the standardized factor loadings varied considerably; many items – particularly in the SE and PA dimensions – showed loadings in the range of <0.2-0.4, suggesting relatively low content representativeness for these items [31]. Correlations between the latent factors were high, indicating potential conceptual overlap between the dimensions. A supplementary exploratory factor analysis (not shown in tables) confirmed this observation: with N=155, a clear four-factor model could not be identified. Furthermore, numerous cross-loadings exceeded 0.32.

The column “MediProf-61” in table 4 (Tab. 4) presents fit indices for the CFA, which overall indicate an inadequate model fit: the RMSEA exceeds the accepted threshold of 0.06, and both the CFI and TLI fall below the recommended values of >0.90 for a good fit [34].

The CFA results reflect the similarly inadequate fit reported by Roos et al. [19] in a small sample with over 60 items. Analogous to that study, table A3.6 in attachment 3 presents a CFA with a substantially reduced number of items. The selection was not based on qualitative criteria but on psychometric properties (see attachment 3 ): items were removed if item-total correlation was <0.3 and/or item difficulty >0.8. The CFA with the remaining 30 items (see table A3.6 in attachment 3 ) showed generally higher standardised loadings, although associations between the latent factors remained pronounced. As in Roos et al. [19], the fit indices improved slightly with the reduced item set (“MediProf-30,” see table 4 (Tab. 4)), but they still did not meet the criteria for an acceptable to good model fit.

### 3.3. Self-assessment of professionalism (research question 2)

Table 5 (Tab. 5) presents the means and standard deviations for the four dimensions of medical professionalism. Overall, students rated their professionalism as high on average. A small proportion of participants (14.8%) rated their professionalism as relatively low (mean>3). 

Considering the individual dimensions, the following percentages of students reported lower self-assessed professionalism:

1. Professionalism towards oneself (SE): 20.8%
2. Professionalism towards patients (PA): 21.1%
3. Professionalism towards other healthcare professions (BG): 12.0%
4. Professionalism towards society (GS): 20.7%

The highest self-assessed professionalism was observed in dimension 3 (“professionalism towards other healthcare professions”), which was rated significantly higher than the other dimensions (p<0.05).

### 3.4. Factors influencing self-assessed professionalism (research question 3)

The regression analysis (see table 6 (Tab. 6)) indicates that none of the collected sociodemographic variables had a significant effect on any of the four dimensions or on the overall professionalism scale (column (5)). The coefficients are small in absolute terms, with correspondingly wide standard errors and 95% confidence intervals. Both R^2^ and F-values are low; the specified models and included variables do not explain the (minor) differences in self-assessed professionalism. This applies particularly to study progression. With one exception, participants did not rate their professionalism significantly higher or lower than first-year students. Students in the Practical Year (6^th^ year) rated their professionalism towards society significantly lower by 0.34 points on the 1-4 scale compared to first-year students. The regression analysis further indicates no overall association between study progression and professionalism; the study year indicators in table 6 (Tab. 6) are not jointly significant (Wald test). Calculations in table 5 (Tab. 5) and table 6 (Tab. 6) were also performed using the maximum possible number of observations per dimension, yielding no differences compared to the sample with fully completed questionnaires (N=155).

### 3.5. Assessment of professionalisation in medical education (research questions 4 and 5)

The survey yielded the following results:

- 94.5% of participants considered the topic of medical professionalism to be very important.
- 79.5% wished for stronger curricular integration of the topic.
- 67.4% indicated that the current curricular implementation at their institution was insufficient.

The percentages refer to the total number of participants in the main quantitative study (N=155). Since both research questions are based on a descriptive analysis of three items, combining study objectives 4 and 5 was considered appropriate. The results indicate a strong interrelation between the perceived importance of professionalisation in medical education and its curricular integration.

## 4. Discussion

### 4.1. Objectives and context

The primary aim of this study was the development and initial psychometric evaluation of the *MediProf *questionnaire for assessing medical professionalism among undergraduate medical students. The study sought to provide preliminary evidence regarding the instrument’s reliability and validity. Additionally, it examined how medical students self-assess their professionalism and the extent to which they desire curricular integration of the topic.

### 4.2. Questionnaire development and psychometric properties

The need for such an instrument arises from the central importance of Professional Identity Formation (PIF) in medical education. The *MediProf *questionnaire was developed based on the *“Professionalism Scale Germany” (Pro-D)* and adapted to the specific needs of medical students. This approach proved successful. On one hand, it was not necessary to develop a completely new questionnaire, as an instrument with good psychometric properties was already available. On the other hand, the iterative adaptation process within the interdisciplinary team allowed for context-specific and target-group-oriented modifications of the items. Analysis of the survey data from medical students provides a first evaluation of the psychometric properties of the *MediProf *questionnaire. Following initial piloting, the instrument demonstrated good reliability overall: internal consistency (considering the varying number of items per dimension) is comparable to the *Pro-D* [19]. Analyses also revealed that some items exhibited low discrimination and high agreement rates. Regarding validity, structural construct validity was assessed using confirmatory factor analysis (CFA). The results indicate that the assumed four-dimensional structure of the questionnaire was only partially reflected. Weak factor loadings in certain dimensions and an overall inadequate model fit point to potential conceptual and empirical weaknesses in the current item structure. These limitations may be related to the relatively small sample size as well as redundant or poorly discriminating items. Further validation with a larger sample and, if necessary, a revised scale structure is therefore required to draw robust conclusions about construct validity. This finding aligns with Roos et al. [19], who found more robust properties in a substantially shortened version of the questionnaire, though they were still unable to fully confirm the theoretical construct of professionalisation.

### 4.3. Self-assessment of professionalism

The surveyed medical students rated their professionalism as high across all dimensions. No influence of age, gender, completed vocational training, prior medical experience, or year of study on self-assessed professionalism across the four dimensions was detected. This finding contradicts the expectation that self-assessed professionalism would increase with study progression and clinical experience. Similar observations have been reported in other studies, suggesting that the development of professional identity is a non-linear, continuous, interactive, and transformative process, shaped by the internalisation of the specific culture of a professional community and influenced by individual, organisational, and interactional factors [35], [36].

### 4.4. Influence of study progression

Notably, students in the Practical Year (PJ) rated their professionalism in dimension 4 (“professionalism towards society”) significantly lower than first-year students. This may indicate that with increasing clinical experience, students develop a deeper understanding and greater awareness of the complexity of professional responsibilities, leading to a more critical self-assessment. It remains to be seen whether this finding would be confirmed in objective measurements or external assessments of professionalism. Conversely, the higher self-assessments in earlier study years may reflect limited experience and a less developed awareness of the challenges of professional practice. Possible explanations include an “overconfidence bias” among first-year students and a more realistic and reflective evaluation by more experienced students. This observation aligns with the Dunning-Kruger effect [37], which describes how individuals with lower expertise tend to overestimate their abilities, whereas increasing expertise leads to a more accurate self-assessment.

### 4.5. Analysis of response patterns by year of study

The underrepresentation of students in the early years of study allows for several interpretations. On one hand, it may indicate that professionalism is not yet perceived as particularly relevant at this stage of the curriculum, with students primarily focused on other aspects such as building a new social environment, adapting learning and time management strategies, or coping with examinations. On the other hand, a lack of awareness regarding the importance of professional identity formation in the early phases of training could have contributed to lower participation. This interpretation is supported by studies [38], [39] emphasizing that fostering professional identity should begin early and continue longitudinally throughout medical education to achieve long-term positive effects on career development. The combination of higher self-assessments in early semesters and a desire for greater curricular integration may reflect an underlying interest and openness to the topic. Students appear willing to engage with professionalism from the outset, highlighting the importance of integrating professionalisation into the curriculum from the first year. Additionally, it is possible that some students did not read the survey invitation or consciously chose not to participate due to lack of interest or motivation.

### 4.6. Methodological considerations

The generally high self-assessment of professionalism, coupled with often uniform response patterns, may partially explain the lack of influence of the examined sociodemographic factors on the outcome variables and the very low variance explained. Another possibility is that additional, unmeasured factors – such as individual personality traits – may play a more substantial role. The sociodemographic parameters included in the analysis primarily capture interindividual variation. The initial CFA suggests a general relationship between the four examined dimensions and their assigned items. However, it also indicates that professionalism among students may need to be distinguished from that among physicians and could be more multidimensional than currently captured by the *MediProf* questionnaire, a hypothesis that should be explored in further data collection. Ordinal regression – such as a cumulative logit model – represents a methodological alternative to the multiple linear regression model used. However, the outcome variables analyzed were based on summed or averaged scores across multiple Likert items per dimension and were therefore treated as quasi-metric. For pragmatic reasons, regarding interpretability and comparability, the linear model was retained, with recognition of its limitations. Preliminary sensitivity analyses using alternative outcome operationalisations (PMAX, factor scores) produced the same results regarding the predictors.

### 4.7. Implications for curricular integration

The newly developed *MediProf* questionnaire was designed as a self-assessment tool to stimulate reflection among medical students while completing the items. Reflective ability is a key prerequisite for professional medical practice and is considered a central method for fostering professionalisation during medical education [40]. Future studies should empirically examine whether and how the use of the questionnaire promotes sustained reflection among students. The context of its application may play a crucial role – for example, within reflection sessions or longitudinal mentoring programs [11]. Qualitative accompanying research could provide further insights into how the questionnaire is used and perceived.

Additionally, the *MediProf *questionnaire could be employed as a formative assessment tool to support students’ professional development. Formative feedback mechanisms are considered central components of competency-based curricula [41], [42] and have long been regarded as essential in medical education [43]. The questionnaire could, for instance, be administered at regular intervals to enable continuous monitoring of professionalisation throughout the course of study.

### 4.8. Limitations

When interpreting the results, it should be noted that the data were collected exclusively at the MHH and therefore cannot be readily generalized to other medical schools. The gender distribution of participants (65% female) corresponds closely to that of enrolled medical students at the MHH (66% female). However, students in the early years of study were underrepresented compared with other surveys at the MHH, suggesting a selective sample. Another methodological limitation concerns the survey dropout rate. Approximately 10% of participants discontinued the survey between dimension 1 (“professionalism towards oneself”) and dimension 2 (“professionalism towards patients”), which aligns with common patterns in online surveys, where participation tends to decline over time [44], [45], [46]. A targeted analysis of dropout rates in future surveys could provide insights for potential optimizations. Furthermore, self-assessments are susceptible to biases such as social desirability and either insufficient or overly critical self-knowledge. No external assessment was included in the current study. Previous research shows that medical students often overestimate their competencies when compared with objective evaluation methods, such as OSCEs [47]. Social desirability may also have contributed to the high self-ratings of professionalism observed. This study represents a first step toward the validation of the *MediProf* questionnaire. Future studies with larger samples and follow-up surveys should aim for additional exploratory and confirmatory factor analyses to further investigate and validate the dimensions of professionalisation among medical students. Moreover, future research should incorporate external assessments alongside self-assessments and validate the questionnaire in diverse contexts.

## 5. Conclusion

The newly developed* MediProf *questionnaire represents the first German-language self-assessment tool for measuring medical students’ professionalism. It demonstrates good psychometric properties in terms of reliability and provides initial evidence of validity. The participants’ expressed desire for stronger curricular integration of the topic underscores the importance of early and continuous promotion of professionalisation throughout medical education.

## 6. Outlook

In the winter semester 2023/24, the MHH introduced the elective course *“Medical identity and professionalisation: Being a physician”*, marking the first offering explicitly focused on the professionalisation of medical students. As part of the course evaluation and to assess learning outcomes, the *MediProf *questionnaire was already applied and will continue to be used in the future.

## Notes

### Data

Data supporting the findings of this scientific publication are available from the corresponding author upon reasonable request.

### Ethical approval

The survey was approved in advance by the Ethics Committee of the MHH (Ref. No. 11195_BO_K_2024). Participation was voluntary and either anonymous or, upon request, pseudonymised. All participants provided informed consent in accordance with the Declaration of Helsinki and the Geneva Declaration. Prior to the survey, students were informed about the nature, purpose, and procedure of the study, as well as data protection measures. They were explicitly advised that they could decline or withdraw their consent at any time without any disadvantages and could contact the project team with any questions.

### Funding

The development of the questionnaire and the cross-sectional survey were conducted as part of the *“Be a Doc”* project, which was funded by the Lower Saxony Ministry of Science and Culture (Call:* “Innovative teaching and learning concepts: Innovation plus”*, Project Number P119, Funding Period: 2023-2024).

### Authors’ ORCIDs

- Stefanos A. Tsikas: [0000-0001-6642-5456]
- Anja Hesse: [0009-0006-3243-0488]
- Kambiz Afshar: [0000-0001-9959-3106]

## Competing interests

The authors declare that they have no competing interests. 

## Supplementary Material

Questionnaire for the assessment of medical professionalism in undergraduate medical education MediProf

Details on the development of the MediProf questionnaire

Test statistics for the four dimensions of the MediProf questionnaire

## Figures and Tables

**Table 1:**
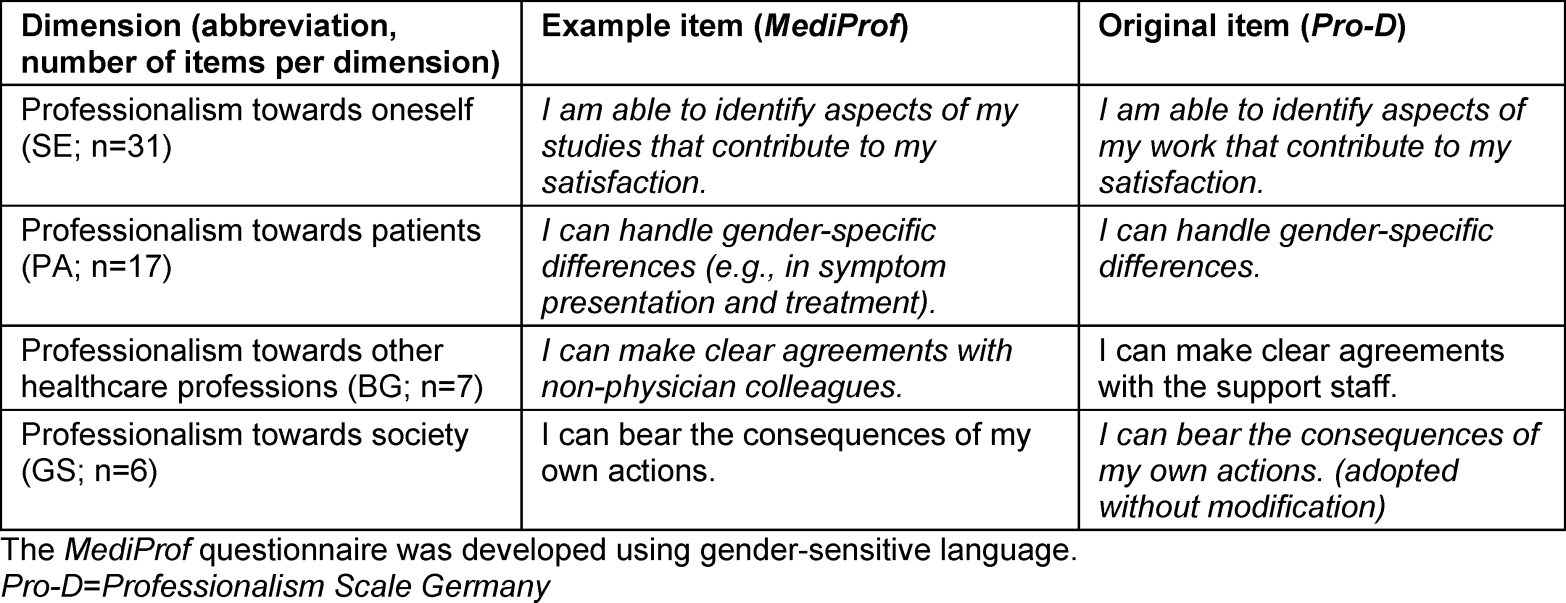
The four dimensions of professionalism in the *MediProf* questionnaire with example items

**Table 2:**
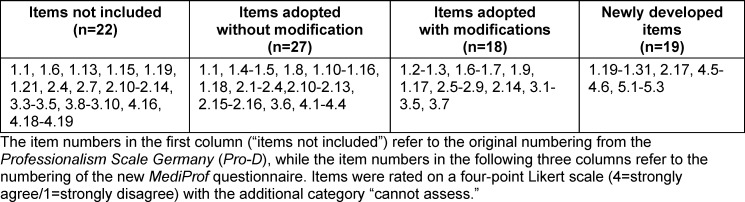
Overview of the composition of the new *MediProf *questionnaire

**Table 3:**
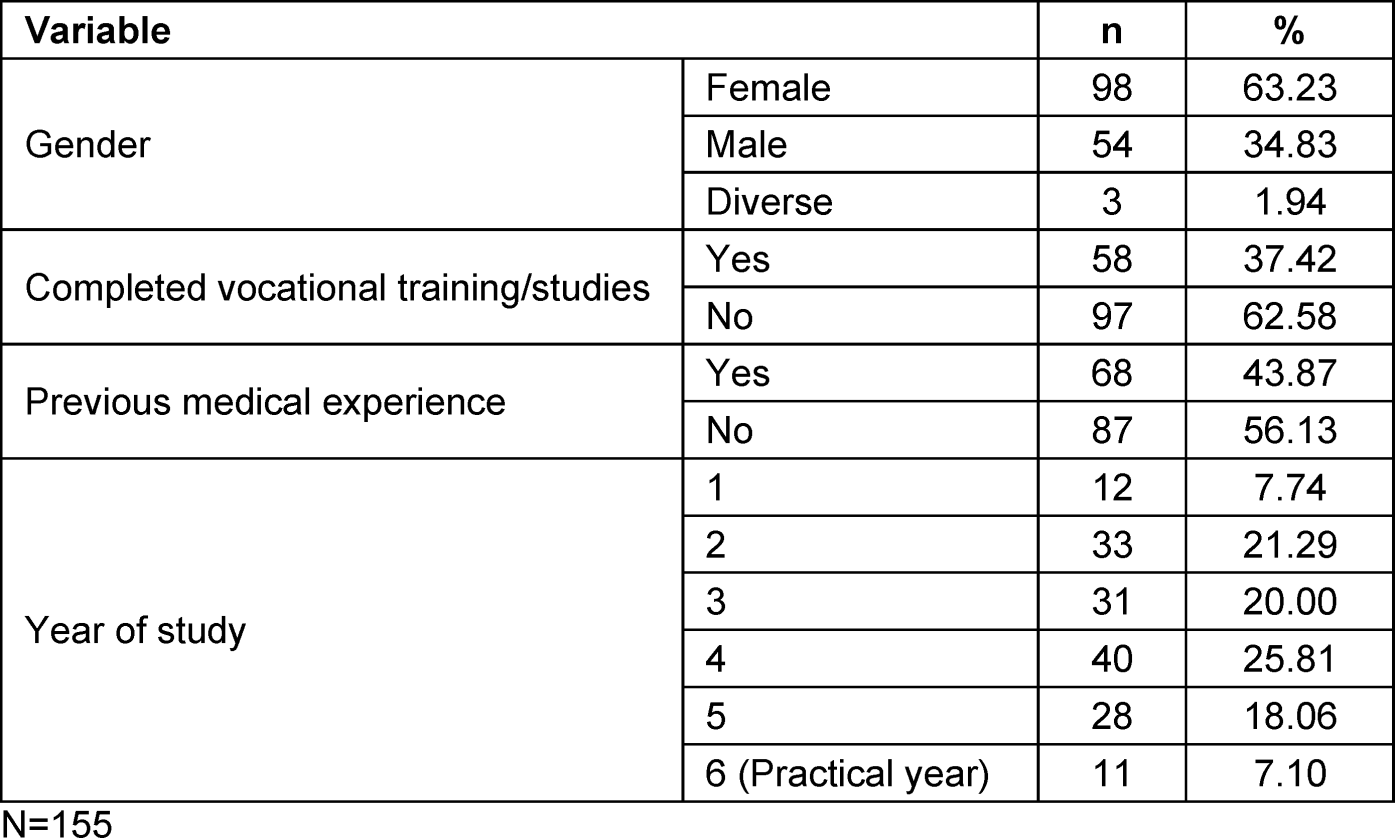
Composition of the sample of participating students

**Table 4:**
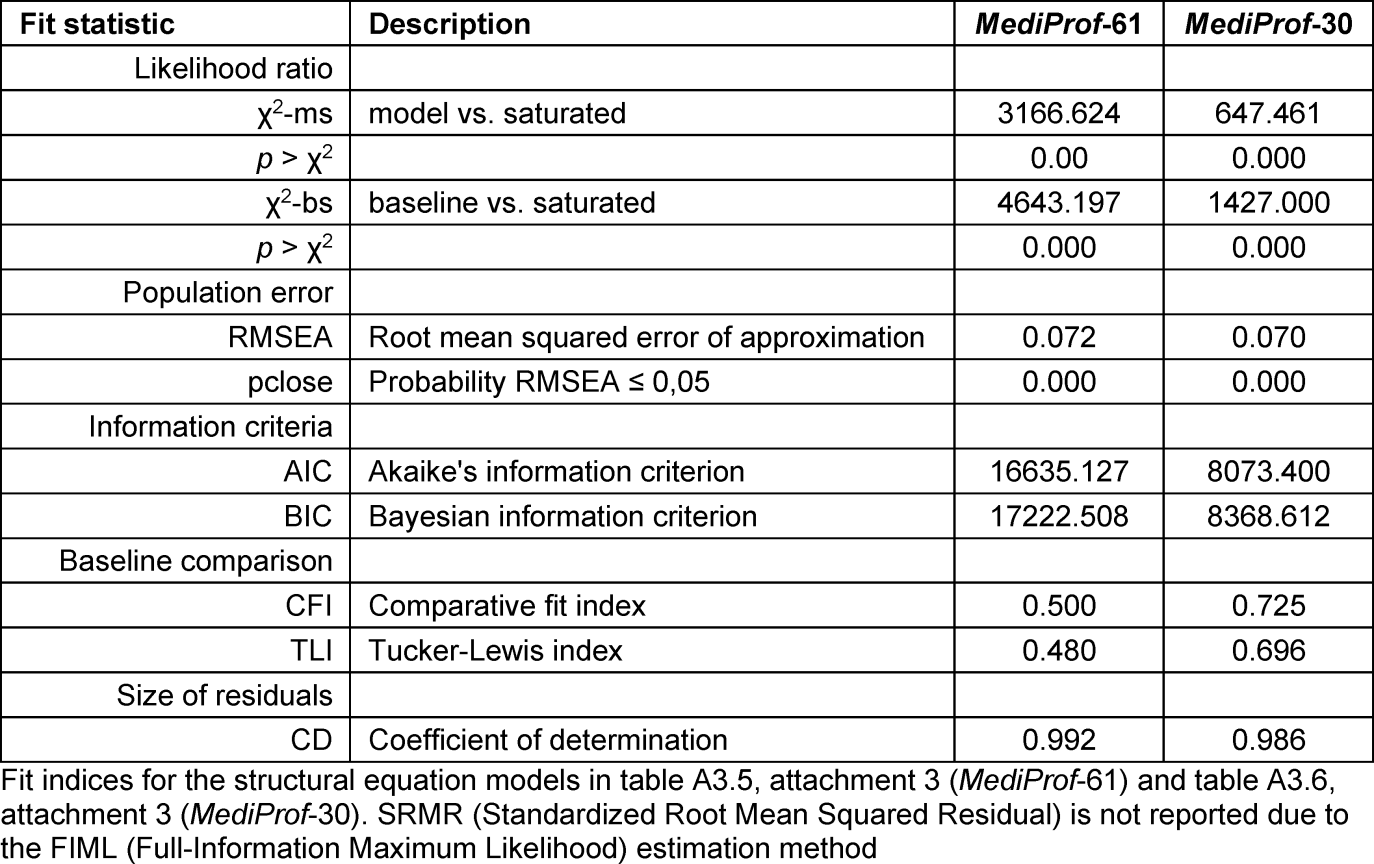
Fit indices for the confirmatory factor analysis

**Table 5:**
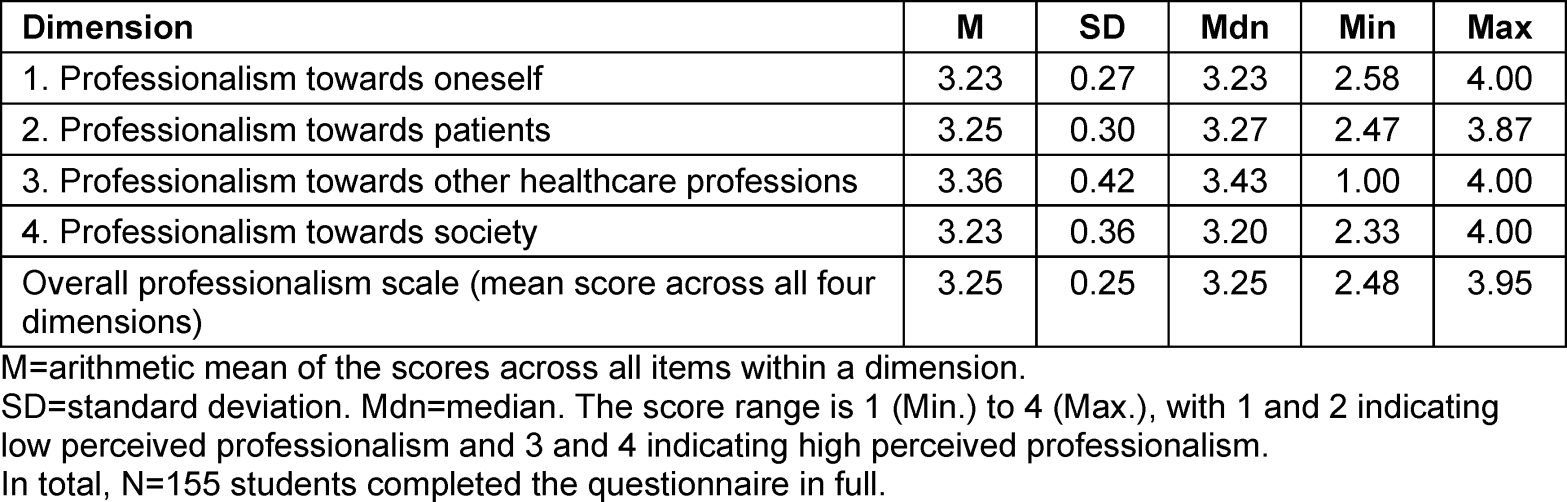
Descriptive statistics per professionalism dimension in the *MediProf* questionnaire

**Table 6:**
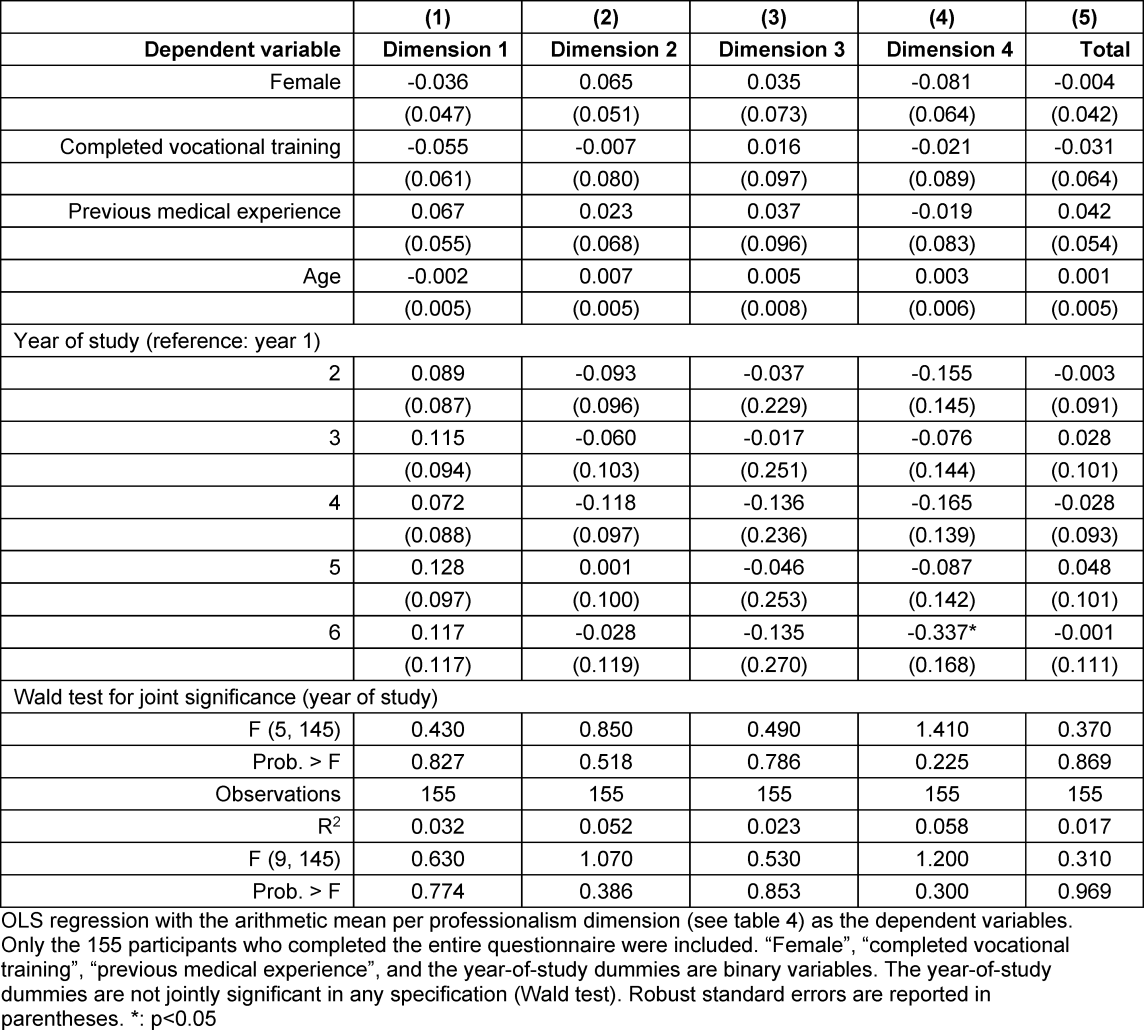
Linear regressions for sociodemographic predictors of self-assessed professionalism in the *MediProf *questionnaire
