# Supplementary material for: Development and psychometric evaluation of an instrument for medical students’ self-assessment of professionalism: Reliability, content, and construct validity of the MediProf questionnaire
Source: GMS J Med Educ. 2026 Mar 23;43(3):Doc40. doi: 10.3205/zma001834 (PMC13054802; doi:10.3205/zma001834)
Supplement: Questionnaire for the assessment of medical professionalism in undergraduate medical education MediProf [file JME-43-40-s-001.pdf]

Attachment 1: Questionnaire for the assessment of medical professionalism in undergraduate medical education MediProf

Attachment 1 to Fey L, Tsikas SA, Meissner S, Hesse A, Afshar K. *Development and psychometric evaluation of an instrument for medical students' self-assessment of professionalism: Reliability, content, and construct validity of the MediProf questionnaire.* GMS J Med Educ. 2026;43(3):Doc40. DOI: 10.3205/zma001834

Questionnaire for the assessment of medical professionalism in undergraduate medical education MediProf

Instructions for completing the questionnaire:

- Please use a ballpoint pen only (do not use a pencil).
- Write any free-text responses in block letters.
- Mark the boxes clearly: ☒
- If you make an incorrect selection by mistake, completely fill in the corresponding box ■ and mark your new choice: ☒

Contact persons:

PD Dr. Kambiz Afshar, MME (Project lead)

Tel.: 0511/532-5855 – afshar.kambiz@mh-hannover.de

Dipl.-Psych. Larissa Fey (Research associate)

Tel.: 0511/532-8599 – fey.larissa@mh-hannover.de

Institute for General Practice and Palliative Care (OE 5440)

Hannover Medical School, Carl-Neuberg-Str. 1, 30625 Hannover

Personal and professional background

How old are you? ______________ (years)

Which gender do you identify with?

☐ female[0] ☐ male[1] ☐ diverse[2]

In which year of study are you currently enrolled? ______________ (year)

Have you already completed vocational training or a degree programme?

☐ yes[1] ☐ no[0]

Please enter your completed vocational training or degree programme here:

Do you have previous medical experience (e.g., working as a paramedic, medical assistant, nurse, etc.)?

☐ yes[1] ☐ no[0]

Please enter your previous medical experience here, if applicable:

Part 1: Professionalism towards oneself

The following statements relate to how you assess your professionalism towards yourself. Please consider situations from your studies.

To what extent do you agree with the following statements?

		Strongly agree[4]	Somewhat agree[3]	Somewhat disagree[2]	Strongly disagree[1]	Cannot assess[0]
1.1	I am able to identify my reactions, thoughts, and feelings elicited by patients.	☐	☐	☐	☐	☐
1.2	I am aware of my professional responsibilities as a physician.	☐	☐	☐	☐	☐
1.3	I use specific situations from my studies as a starting point for critical self-reflection.	☐	☐	☐	☐	☐
1.4	I discuss bottlenecks and mistakes without losing confidence in my own competence.	☐	☐	☐	☐	☐
1.5	I have a realistic assessment of my strengths and weaknesses.	☐	☐	☐	☐	☐
1.6	I am able to maintain a balance between my studies and personal life.	☐	☐	☐	☐	☐
1.7	I am able to identify aspects of my studies that contribute to my satisfaction.	☐	☐	☐	☐	☐
1.8	I am able to cope with the possibility that a fundamental decision I make may not be successful.	☐	☐	☐	☐	☐
1.9	I take suggestions from feedback discussions into account.	☐	☐	☐	☐	☐
1.10	I am interested in others' opinions about my behaviour.	☐	☐	☐	☐	☐
1.11	I allow myself to be evaluated.	☐	☐	☐	☐	☐
1.12	I am eager to learn (I ask questions and take initiative).	☐	☐	☐	☐	☐
1.13	I am able to admit mistakes.	☐	☐	☐	☐	☐
1.14	I take proactive steps to correct mistakes I have made.	☐	☐	☐	☐	☐
1.15	I do not avoid the consequences of a mistake I have made.	☐	☐	☐	☐	☐
1.16	I quickly regain my composure after an unpleasant conversation.	☐	☐	☐	☐	☐
1.17	I am able to cope with challenging situations.	☐	☐	☐	☐	☐

		Strongly agree[4]	Somewhat agree[3]	Somewhat disagree[2]	Strongly disagree[1]	Cannot assess[0]
1.18	I am able to express my own opinion clearly and confidently.	☐	☐	☐	☐	☐
1.19	I reflect on what constitutes good medical practice.	☐	☐	☐	☐	☐
1.20	The further I progress in medical school, the more confident I feel in my role as a physician.	☐	☐	☐	☐	☐
1.21	I am able to handle criticism constructively.	☐	☐	☐	☐	☐
1.22	I reflect on the kind of physician I want to be.	☐	☐	☐	☐	☐
1.23	The further I progress in medical school, the more I notice the knowledge gap between myself and a layperson.	☐	☐	☐	☐	☐
1.24	I am aware of my limits and can take them into account in my medical practice.	☐	☐	☐	☐	☐
1.25	I know which knowledge gaps I still want to close during my medical studies.	☐	☐	☐	☐	☐
1.26	I know how to remain calm in challenging situations.	☐	☐	☐	☐	☐
1.27	I understand my medical role as one of lifelong learning.	☐	☐	☐	☐	☐
1.28	I take time to reflect on myself and my medical practice.	☐	☐	☐	☐	☐
1.29	Physician role models are important for my own medical practice.	☐	☐	☐	☐	☐
1.30	Negative role models help me understand how I do not want to act in my medical role.	☐	☐	☐	☐	☐
1.31	I take positive physician role models as examples for shaping my own professional identity.	☐	☐	☐	☐	☐

Part 2: Professionalism towards patients

The following statements relate to how you assess your professionalism towards patients. If you have not yet had contact with patients, please select the response option “Cannot assess.”

To what extent do you agree with the following statements?

		Strongly agree[4]	Somewhat agree[3]	Somewhat disagree[2]	Strongly disagree[1]	Cannot assess[0]
2.1	I am able to address difficult topics.	☐	☐	☐	☐	☐
2.2	I respect patients’ right to have a say in their care.	☐	☐	☐	☐	☐
2.3	I am able to express my compassion.	☐	☐	☐	☐	☐
2.4	I am able to deal with patients’ feelings of shame.	☐	☐	☐	☐	☐
2.5	I am unbiased toward all patients (e.g., regarding their sexual orientation, social status, or religion).	☐	☐	☐	☐	☐
2.6	Maintaining a neat appearance is an especially important part of my professional conduct.	☐	☐	☐	☐	☐
2.7	I can adapt my language appropriately to the language and understanding of my patients.	☐	☐	☐	☐	☐
2.8	I can handle gender-specific differences (e.g., in symptom presentation and treatment).	☐	☐	☐	☐	☐
2.9	I can handle different patient expectations regarding medical care.	☐	☐	☐	☐	☐
2.10	I incorporate the medical history of my patients into their care.	☐	☐	☐	☐	☐
2.11	I take the social situation of my patients into account in their care.	☐	☐	☐	☐	☐
2.12	I can respond to life events of my patients when necessary.	☐	☐	☐	☐	☐
2.13	I respect the self-determination of my patients.	☐	☐	☐	☐	☐
2.14	I handle professional confidentiality carefully in conversations with fellow students and acquaintances.	☐	☐	☐	☐	☐
2.15	I can separate myself from patients’ emotions.	☐	☐	☐	☐	☐
2.16	I can take interventions that reduce patients’ aggression.	☐	☐	☐	☐	☐
2.17	I reflect on the possibility that, in my future role as a physician, I may no longer show empathy toward patients because many things have become normalized for me over time.	☐	☐	☐	☐	☐

Part 3: Professionalism towards other healthcare professions

The following statements relate to how you assess your professionalism towards other healthcare professions. Please consider situations both during your studies and in clinical settings, such as on the ward or in practice.

To what extent do you agree with the following statements?

		Strongly agree[4]	Somewhat agree[3]	Somewhat disagree[2]	Strongly disagree[1]	Cannot assess[0]
3.1	I consult professionals from other occupational groups with focused questions.	☐	☐	☐	☐	☐
3.2	I ensure a structured exchange with professionals from other occupational groups.	☐	☐	☐	☐	☐
3.3	I behave appropriately towards individuals from other professional groups.	☐	☐	☐	☐	☐
3.4	I can make clear agreements with non-physician colleagues.	☐	☐	☐	☐	☐
3.5	I am open to suggestions from non-physician colleagues.	☐	☐	☐	☐	☐
3.6	I can address problems in collaboration with others immediately.	☐	☐	☐	☐	☐
3.7	I can handle conflicts in the team constructively.	☐	☐	☐	☐	☐

Part 4: Professionalism towards society

The following statements relate to how you assess your professionalism towards society.
To what extent do you agree with the following statements?

		Strongly agree^{4}	Somewhat agree^{3}	Somewhat disagree^{2}	Strongly disagree^{1}	Cannot assess^{0}
4.1	I can bear the consequences of my own actions.	☐	☐	☐	☐	☐
4.2	I can take responsibility for deviating from rules and guidelines.	☐	☐	☐	☐	☐
4.3	I can justify a decision I make based on scientific evidence.	☐	☐	☐	☐	☐
4.4	I can explain my own norms and values regarding the use of scientific evidence.	☐	☐	☐	☐	☐
4.5	I align my medical practice with specific values and norms.	☐	☐	☐	☐	☐
4.6	I can distinguish between private and professional contexts or roles.	☐	☐	☐	☐	☐

Part 5: Professionalisation in medical education

Finally, we would like to know how you experience the topic of “professional development” in relation to your university.

To what extent do you agree with the following statements?

		Strongly agree[4]	Somewhat agree[3]	Somewhat disagree[2]	Strongly disagree[1]	Cannot assess[0]
5.1	I consider the topic of professionalisation in medical education to be very important.	☐	☐	☐	☐	☐
5.2	I wish that my university would address the topic of professionalisation in medical education even more intensively.	☐	☐	☐	☐	☐
5.3	I think that my university has already sufficiently integrated the topic of professionalisation in medical education.	☐	☐	☐	☐	☐
