# Supplementary material for: Development and psychometric evaluation of an instrument for medical students’ self-assessment of professionalism: Reliability, content, and construct validity of the MediProf questionnaire
Source: GMS J Med Educ. 2026 Mar 23;43(3):Doc40. doi: 10.3205/zma001834 (PMC13054802; doi:10.3205/zma001834)
Supplement: Details on the development of the MediProf questionnaire [file JME-43-40-s-002.pdf]

Attachment 2: Details on the development of the *MediProf* questionnaire

Table A2.1: Overview of *MediProf* questionnaire iems compared with the *Professionalism Scale Germany (Pro-D)* items, including details on the adaptation process

Item no.	Item description in the *MediProf* questionnaire	Item no.	Item description in the *Pro-D* questionnaire	Notes
**Part 1: Professionalism towards oneself**				
1.1	I am able to identify my reactions, thoughts, and feelings elicited by patients.	4.1	I am able to identify my reactions, thoughts, and feelings elicited by patients.	Adopted without modification
1.2	I am aware of my professional responsibilities as a physician.	3.1	I am able to bear the consequences of my own actions.	Adopted with modifications
1.3	I use specific situations from my studies as a starting point for critical self-reflection.	4.3	I use specific clinical situations as a starting point for critical self-reflection.	Adopted with modifications
1.4	I discuss bottlenecks and mistakes without losing confidence in my own competence.	4.4	I discuss bottlenecks and mistakes without losing confidence in my own competence.	Adopted without modification
1.5	I have a realistic assessment of my strengths and weaknesses.	4.5	I have a realistic assessment of my strengths and weaknesses.	Adopted without modification
1.6	I am able to maintain a balance between my studies and personal life.	4.6	I am able to maintain a balance between work and personal life.	Adopted with modifications
1.7	I am able to identify aspects of my studies that contribute to my satisfaction.	4.7	I am able to identify aspects of my work that contribute to my satisfaction.	Adopted with modifications
1.8	I am able to cope with the possibility that a fundamental decision I make may not be successful.	4.8	I am able to cope with the possibility that a fundamental decision I make may not be successful.	Adopted without modification
1.9	I take suggestions from feedback discussions into account.	4.9	I follow through on agreements made during feedback discussions.	Adopted with modifications
1.10	I am interested in others' opinions about my behaviour.	4.10	I am interested in others' opinions about my behaviour.	Adopted without modification
1.11	I allow myself to be evaluated.	4.11	I allow myself to be evaluated.	Adopted without modification
1.12	I am eager to learn (I ask questions and take initiative).	4.12	I am eager to learn (I ask questions and take initiative).	Adopted without modification
1.13	I am able to admit mistakes.	4.13	I am able to admit mistakes.	Adopted without modification
1.14	I take proactive steps to correct mistakes I have made.	4.14	I take proactive steps to correct mistakes I have made.	Adopted without modification
1.15	I do not avoid the consequences of a mistake I have made.	4.15	I do not avoid the consequences of a mistake I have made.	Adopted without modification
1.16	I quickly regain my composure after an unpleasant conversation.	4.17	I quickly regain my composure after an unpleasant conversation.	Adopted without modification
1.17	I am able to cope with challenging situations.	4.20	I am able to deal with difficult patients.	Adopted with modifications
1.18	I am able to express my own opinion clearly and confidently.	4.22	I am able to express my own opinion clearly and confidently.	Adopted without modification
1.19	I reflect on what constitutes good medical practice.			Newly developed
1.20	The further I progress in medical school, the more confident I feel in my role as a physician.			Newly developed

1.21	I am able to handle criticism constructively.			Newly developed
1.22	I reflect on the kind of physician I want to be.			Newly developed
1.23	The further I progress in medical school, the more I notice the knowledge gap between myself and a layperson.			Newly developed
1.24	I am aware of my limits and can take them into account in my medical practice.			Newly developed
1.25	I know which knowledge gaps I still want to close during my medical studies.			Newly developed
1.26	I know how to remain calm in challenging situations.			Newly developed
1.27	I understand my medical role as one of lifelong learning.			Newly developed
1.28	I take time to reflect on myself and my medical practice.			Newly developed
1.29	Physician role models are important for my own medical practice.			Newly developed
1.30	Negative role models help me understand how I do not want to act in my medical role.			Newly developed
1.31	I take positive physician role models as examples for shaping my own professional identity.			Newly developed
**Part 2: Professionalism towards patients**				
2.1	I am able to address difficult topics.	1.2	I am able to address difficult topics.	Adopted without modification
2.2	I respect patients' right to have a say in their care.	1.3	I respect patients' right to have a say in their care.	Adopted without modification
2.3	I am able to express my compassion.	1.4	I am able to express my compassion.	Adopted without modification
2.4	I am able to deal with patients' feelings of shame.	1.5	I am able to deal with patients' feelings of shame.	Adopted without modification
2.5	I am unbiased toward all patients (e.g., regarding their sexual orientation, social status, or religion).	1.7	I am unbiased toward patients from different socio-cultural backgrounds (e.g., religion).	Adopted with modifications
2.6	Maintaining a neat appearance is an especially important part of my professional conduct.	1.8	I maintain a neat personal appearance.	Adopted with modifications
2.7	I can adapt my language appropriately to the language and understanding of my patients.	1.9	I can adapt my language to match the patient's language.	Adopted with modifications
2.8	I can handle gender-specific differences (e.g., in symptom presentation and treatment).	1.10	I can handle gender-specific differences.	Adopted with modifications
2.9	I can handle different patient expectations regarding medical care.	1.11	I can handle different patient expectations of a general practitioner.	Adopted with modifications
2.10	I incorporate the medical history of my patients into their care.	1.12	I incorporate the medical history of my patients into their care.	Adopted without modification
2.11	I take the social situation of my patients into account in their care.	1.14	I take the social situation of my patients into account in their care.	Adopted without modification
2.12	I can respond to life events of my patients when necessary.	1.16	I can respond to life events of my patients when necessary.	Adopted without modification
2.13	I respect the self-determination of my patients.	1.17	I respect the self-determination of my patients.	Adopted without modification

2.14	I handle professional confidentiality carefully in conversations with fellow students and acquaintances.	1.18	I handle professional confidentiality carefully in conversations with close contacts and acquaintances.	Adopted with modifications
2.15	I can separate myself from patients' emotions.	1.20	I can separate myself from patients' emotions.	Adopted without modification
2.16	I can take interventions that reduce patients' aggression.	4.24	I can take interventions that reduce patients' aggression.	Adopted without modification
2.17	I reflect on the possibility that, in my future role as a physician, I may no longer show empathy toward patients because many things have become normalized for me over time.			Newly developed
**Part 3: Professionalism towards other healthcare professions**				
3.1	I consult professionals from other occupational groups with focused questions.	2.1	I consult other medical professionals with focused questions.	Adopted with modifications
3.2	I ensure a structured exchange with professionals from other occupational groups.	2.2	I ensure a structured exchange with other medical groups.	Adopted with modifications
3.3	I behave appropriately towards individuals from other professional groups.	2.3	I maintain appropriate conduct when responding to targeted questions from other medical groups.	Adopted with modifications
3.4	I can make clear agreements with non-physician colleagues.	2.5	I can make clear agreements with the support staff.	Adopted with modifications
3.5	I am open to suggestions from non-physician colleagues.	2.6	I respond openly to contributions from the support staff.	Adopted with modifications
3.6	I can address problems in collaboration with others immediately.	2.8	I can address problems in collaboration with others immediately.	Adopted without modification
3.7	I can handle conflicts in the team constructively.	2.9	I can handle conflicts constructively.	Adopted with modifications
**Part 4: Professionalism towards society**				
4.1	I can bear the consequences of my own actions.	3.1	I can bear the consequences of my own actions.	Adopted without modification
4.2	I can take responsibility for deviating from rules and guidelines.	3.2	I can take responsibility for deviating from rules and guidelines.	Adopted without modification
4.3	I can justify a decision I make based on scientific evidence.	3.6	I can justify a decision I make based on scientific evidence.	Adopted without modification
4.4	I can explain my own norms and values regarding the use of scientific evidence.	3.7	I can explain my own norms and values regarding the use of scientific evidence.	Adopted without modification
4.5	I align my medical practice with specific values and norms.			Newly developed
4.6	I can distinguish between private and professional contexts or roles.			Newly developed
**Part 5: Professionalisation in medical education**				
5.1	I consider the topic of professionalisation in medical education to be very important.			Newly developed
5.2	I wish that my university would address the topic of professionalisation in medical education even more intensively.			Newly developed
5.3	I think that my university has already sufficiently integrated the topic of professionalisation in medical education.			Newly developed

Table A2.2: Overview of Items from the *Professionalism Scale Germany (Pro-D)* not adopted (n=22)

Item no.	Description
1.1	I can provide patient information according to legal requirements while considering patient preferences.
1.6	I explain the procedure and purpose of physical examinations to patients.
1.13	I take into account the impact of political frameworks on my patients.
1.15	I actively include the medical history (e.g., chronic illnesses) in regular care (e.g., DMP).
1.19	I do not give patients false hope.
1.21	I am not influenced by the social status of patients.
2.4	I can motivate supporting staff.
2.7	I accurately convey instructions.
2.10	I can manage the boundaries between primary care physicians and specialists.
2.11	I can ensure coordinated medical care in primary and specialized care.
2.12	I can distinguish between personal and professional matters in negotiations.
2.13	I can make business-related decisions.
2.14	I can conduct an employee discussion.
3.3	I can assess the influence of my own norms regarding illness on my medical actions.
3.4	I am aware of the importance and relative value of scientific evidence in decision-making.
3.5	I weigh legal regulations against factors related to the patient and their circumstances.
3.8	I can identify suboptimal care in practice.
3.9	I can develop a quality improvement project.
3.10	I can justify the indication for a home visit.
4.16	I adapt and remain composed when patients need to be seen unexpectedly.
4.18	I allow minor illnesses (e.g., fatigue) to take their natural course, even if the diagnosis is unclear.
4.19	I can process my own mistakes.
