# Supplementary material for: Development and psychometric evaluation of an instrument for medical students’ self-assessment of professionalism: Reliability, content, and construct validity of the MediProf questionnaire
Source: GMS J Med Educ. 2026 Mar 23;43(3):Doc40. doi: 10.3205/zma001834 (PMC13054802; doi:10.3205/zma001834)
Supplement: Test statistics for the four dimensions of the MediProf questionnaire [file JME-43-40-s-003.pdf]

Attachment 3: Test statistics for the four dimensions of the *MediProf* questionnaire

The content of the individual items can be found in the *MediProf* questionnaire in attachment 1.

SE02: Dimension “Professionalism towards oneself”
PA01: Dimension “Professionalism towards patients”
BG01: Dimension “Professionalism towards other healthcare professions”
GS01: Dimension “Professionalism towards society”

Item	N	Item-Test Correlation	Item-Rest Correlation	Average inter-item covariance	Alpha	Item difficulty
SE02_01	153	0.4073	0.3487	0.0638	0.8336	0.8061
SE02_02	154	0.4160	0.3622	0.0638	0.8333	0.8766
SE02_03	151	0.4725	0.4114	0.0625	0.8314	0.7859
SE02_04	153	0.4079	0.3367	0.0631	0.8337	0.5556
SE02_05	149	0.3843	0.3214	0.0638	0.8342	0.6577
SE02_06	154	0.3851	0.2904	0.0626	0.8364	0.5823
SE02_07	155	0.4896	0.4084	0.0611	0.8313	0.6667
SE02_08	141	0.3907	0.3189	0.0631	0.8341	0.6028
SE02_09	153	0.4336	0.3762	0.0634	0.8327	0.8083
SE02_10	154	0.3231	0.2543	0.0644	0.8362	0.8009
SE02_11	152	0.4769	0.4185	0.0627	0.8314	0.7610
SE02_12	154	0.3046	0.2395	0.0648	0.8365	0.8615
SE02_13	154	0.3697	0.3052	0.0640	0.8348	0.7835
SE02_14	155	0.4429	0.3933	0.0637	0.8327	0.8581
SE02_15	154	0.4598	0.3837	0.0620	0.8323	0.7273
SE02_16	154	0.4544	0.3737	0.0618	0.8325	0.5303
SE02_17	154	0.3912	0.3363	0.0641	0.8340	0.7359
SE02_18	154	0.2757	*0.2011*	0.0649	0.8379	0.7597
SE02_19	154	0.4197	0.3512	0.0630	0.8331	0.7597
SE02_20	148	0.5227	0.4453	0.0605	0.8297	0.5968
SE02_21	155	0.5221	0.4726	0.0626	0.8303	0.7505
SE02_22	155	0.4773	0.4154	0.0624	0.8313	0.8624
SE02_23	155	0.5165	0.4635	0.0625	0.8304	0.8753
SE02_24	153	0.3288	0.2498	0.0640	0.8367	0.5969
SE02_25	154	0.5263	0.4556	0.0609	0.8295	0.7100
SE02_26	154	0.3643	0.2850	0.0635	0.8356	0.6602
SE02_27	155	0.3998	0.3435	0.0639	0.8337	0.8903
SE02_28	152	0.5583	0.4985	0.0610	0.8283	0.6864
SE02_29	153	0.3206	*0.2225*	0.0636	0.8388	0.7777
SE02_30	153	0.2821	*0.2089*	0.0649	0.8376	0.8802
SE02_31	154	0.4061	0.3474	0.0637	0.8334	0.8701
				0.0631	**0.8380**	0.7444

Notes: N: Number of completed responses (max.: 155). Item–total correlation: correlation with the total score including the item itself. Item–rest correlation (i.e., discrimination): correlation with the total score excluding the respective item. Alpha: Cronbach’s alpha if the item is deleted. Items with low discrimination are shown in italics.

Table A3.1: Questionnaire test statistics for the dimension “*professionalism towards oneself*”

Item	N	Item-Test Correlation	Item-Rest Correlation	Average inter-item covariance	Alpha	Item difficulty
PA01_01	148	0.3322	*0.2103*	0.0695	0.7448	0.6892
PA01_02	154	0.3813	0.2783	0.0692	0.7398	0.8896
PA01_03	155	0.4567	0.3356	0.0664	0.7355	0.7871
PA01_04	154	0.4888	0.3764	0.0657	0.7315	0.7749
PA01_05	153	0.5024	0.3877	0.0648	0.7303	0.8170
PA01_06	155	0.3843	0.2612	0.0686	0.7415	0.7849
PA01_07	155	0.4744	0.3698	0.0660	0.7314	0.8172
PA01_08	138	0.6355	0.5333	0.0603	0.7159	0.6957
PA01_09	146	0.4896	0.3838	0.0655	0.7305	0.6804
PA01_10	137	0.6056	0.5364	0.0645	0.7231	0.8175
PA01_11	142	0.5550	0.4607	0.0639	0.7248	0.7911
PA01_12	135	0.6398	0.5532	0.0609	0.7153	0.7506
PA01_13	153	0.3959	0.3101	0.0695	0.7383	0.9129
PA01_14	154	0.3905	0.2447	0.0672	0.7422	0.8052
PA01_15	155	0.2027	*0.0464*	0.0736	0.7628	0.6581
PA01_16	127	0.5095	0.3833	0.0633	0.7297	0.5328
PA01_17	149	0.3685	*0.1656*	0.0684	0.7598	0.5123
				0.0663	**0.7471**	0.7480
Notes: N: Number of completed responses (max.: 155). Item–total correlation: correlation with the total score including the item itself. Item–rest correlation (i.e., discrimination): correlation with the total score excluding the respective item. Alpha: Cronbach's alpha if the item is deleted. Items with low discrimination are shown in italics.						

Table A3.2: Questionnaire test statistics for the dimension "*professionalism towards patients*"

Item	N	Item-Test Correlation	Item-Rest Correlation	Average inter-item covariance	Alpha	Item difficulty
BG01_01	146	0.6430	0.4690	0.1351	0.7484	0.7922
BG01_02	139	0.7084	0.5349	0.1215	0.7313	0.7242
BG01_03	154	0.6444	0.5136	0.1397	0.7385	0.9091
BG01_04	147	0.6715	0.5436	0.1352	0.7321	0.8844
BG01_05	152	0.5598	0.4021	0.1470	0.7561	0.8991
BG01_06	149	0.6649	0.4637	0.1270	0.7518	0.6286
BG01_07	145	0.7032	0.5263	0.1227	0.7357	0.6713
				0.1326	0.7705	0.7870
Notes: N: Number of completed responses (max.: 155). Item–total correlation: correlation with the total score including the item itself. Item–rest correlation (i.e., discrimination): correlation with the total score excluding the respective item. Alpha: Cronbach's alpha if the item is deleted. Items with low discrimination are shown in italics.						

Table A3.3: Questionnaire test statistics for the dimension "*professionalism towards other healthcare professions*"

Item	N	Item-Test Correlation	Item-Rest Correlation	Average inter-item covariance	Alpha	Item difficulty
GS01_01	152	0.5668	0.3063	0.0818	0.5763	0.7697
GS01_02	128	0.5310	*0.2044*	0.0863	0.6261	0.6224
GS01_03	135	0.7228	0.5289	0.0607	0.4858	0.6938
GS01_04	131	0.6341	0.4047	0.0713	0.5384	0.7481
GS01_05	152	0.5401	0.3160	0.0888	0.5874	0.8180
GS01_06	152	0.5533	0.2617	0.0857	0.6001	0.7807
				0.0790	0.6147	0.7388
Notes: N: Number of completed responses (max.: 155). Item–total correlation: correlation with the total score including the item itself. Item–rest correlation (i.e., discrimination): correlation with the total score excluding the respective item. Alpha: Cronbach's alpha if the item is deleted. Items with low discrimination are shown in italics.						

Table A3.4: Questionnaire test statistics for the dimension "*professionalism towards society*"

	Coef.	OIM SE	var (ε)	OIM SE
**SE (Professionalism towards oneself)**				
SE02_01	0.393*	0.075	0.846	0.059
SE02_02	0.388*	0.075	0.850	0.058
SE02_03	0.435*	0.074	0.810	0.064
SE02_04	0.364*	0.077	0.867	0.056
SE02_05	0.336*	0.080	0.887	0.054
SE02_06	0.301*	0.080	0.909	0.048
SE02_07	0.423*	0.073	0.821	0.062
SE02_08	0.359*	0.078	0.871	0.056
SE02_09	0.431*	0.073	0.814	0.063
SE02_10	0.275*	0.081	0.924	0.045
SE02_11	0.438*	0.072	0.808	0.063
SE02_12	0.228*	0.083	0.948	0.038
SE02_13	0.357*	0.078	0.872	0.055
SE02_14	0.431*	0.072	0.814	0.062
SE02_15	0.443*	0.072	0.803	0.064
SE02_16	0.401*	0.076	0.839	0.061
SE02_17	0.370*	0.077	0.863	0.057
SE02_18	0.248*	0.083	0.939	0.041
SE02_19	0.363*	0.078	0.869	0.057
SE02_20	0.441*	0.073	0.805	0.064
SE02_21	0.538*	0.065	0.711	0.070
SE02_22	0.441*	0.073	0.806	0.064
SE02_23	0.480*	0.069	0.770	0.067
SE02_24	0.287*	0.081	0.918	0.046
SE02_25	0.534*	0.065	0.715	0.070
SE02_26	0.303*	0.080	0.908	0.049
SE02_27	0.414*	0.074	0.829	0.061
SE02_28	0.560*	0.063	0.686	0.071
SE02_29	0.239*	0.083	0.943	0.040
SE02_30	0.217*	0.084	0.953	0.036
SE02_31	0.362*	0.077	0.869	0.056

**PA (Professionalism towards patients)**				
PA01_01	0.264*	0.085	0.930	0.045
PA01_02	0.336*	0.082	0.887	0.055
PA01_03	0.402*	0.079	0.839	0.063
PA01_04	0.374*	0.080	0.860	0.060
PA01_05	0.375*	0.079	0.859	0.059
PA01_06	0.310*	0.082	0.904	0.051
PA01_07	0.467*	0.073	0.781	0.068
PA01_08	0.655*	0.059	0.571	0.078
PA01_09	0.500*	0.072	0.750	0.072
PA01_10	0.549*	0.069	0.699	0.076
PA01_11	0.514*	0.071	0.736	0.073
PA01_12	0.660*	0.059	0.565	0.078
PA01_13	0.327*	0.082	0.893	0.054
PA01_14	0.256*	0.084	0.935	0.043
PA01_15	0.140*	0.089	0.980	0.025
PA01_16	0.459*	0.079	0.790	0.072
PA01_17	0.196*	0.089	0.962	0.035
**BG (Professionalism towards other healthcare professions)**				
BG01_01	0.551*	0.069	0.696	0.076
BG01_02	0.617*	0.066	0.619	0.081
BG01_03	0.668*	0.057	0.554	0.077
BG01_04	0.723*	0.053	0.477	0.077
BG01_05	0.565*	0.066	0.680	0.075
BG01_06	0.417*	0.077	0.826	0.064
BG01_07	0.481*	0.073	0.769	0.070
**GS (Professionalism towards society)**				
GS01_01	0.528*	0.085	0.722	0.090
GS01_02	0.249*	0.103	0.938	0.051
GS01_03	0.545*	0.108	0.703	0.118
GS01_04	0.447*	0.119	0.800	0.106
GS01_05	0.408*	0.088	0.834	0.071
GS01_06	0.537*	0.090	0.712	0.096
	Coef.	OIM SE		
cov(ε.SE02_10*ε.SE02_11)	0.490*	0.064		
cov(ε.SE02_29*ε.SE02_31)	0.502*	0.062		
cov(ε.PA01_10*ε.PA01_11)	0.681*	0.049		
cov(ε.BG01_06*ε.BG01_07)	0.588*	0.056		
cov(SE02*PA01)	0.618*	0.072		
cov(SE02*BG01)	0.660*	0.068		
cov(SE02*GS01)	0.626*	0.097		
cov(PA01*BG01)	0.723*	0.065		
cov(PA01*GS01)	0.645*	0.098		
cov(BG01*GS01)	0.586*	0.119		
Notes: SEM output of the confirmatory factor analysis including all *MediProf* items and the four questionnaire dimensions as latent, standardised (= 1) factors. Full Information Maximum Likelihood (FIML) estimation was used. Covariances between error terms were included based on modification indices (Lagrange Multiplier tests) to improve model fit. SE: standard error. N = 155. LR test (model vs. saturated): χ^2 (1759) = 3166.22; Prob > χ^2: $p < 0.001$. *: $p < 0.05$.				

Table A3.5: Confirmatory factor analysis (all *MediProf* items)

	Coef.	OIM SE	var (ε)	OIM SE
**SE (Professionalism towards oneself)**				
SE02_03	0.312*	0.084	0.903	0.053
SE02_04	0.441*	0.075	0.805	0.067
SE02_05	0.370*	0.081	0.863	0.060
SE02_07	0.300*	0.084	0.910	0.050
SE02_08	0.410*	0.079	0.832	0.064
SE02_11	0.477*	0.073	0.773	0.070
SE02_13	0.424*	0.078	0.821	0.066
SE02_15	0.391*	0.079	0.847	0.062
SE02_16	0.565*	0.068	0.681	0.077
SE02_17	0.435*	0.077	0.811	0.067
SE02_19	0.242*	0.088	0.942	0.042
SE02_20	0.357*	0.082	0.873	0.059
SE02_21	0.690*	0.056	0.524	0.077
SE02_25	0.373*	0.082	0.861	0.061
SE02_28	0.494*	0.073	0.756	0.072
**PA (Professionalism towards patients)**				
PA01_03	0.394*	0.082	0.844	0.065
PA01_04	0.393*	0.083	0.846	0.065
PA01_08	0.742*	0.058	0.449	0.086
PA01_09	0.434*	0.079	0.811	0.069
PA01_11	0.518*	0.074	0.732	0.077
PA01_12	0.686*	0.063	0.529	0.086
PA01_16	0.454*	0.083	0.794	0.076
**BG (Professionalism towards other healthcare professions)**				
BG01_01	0.343*	0.089	0.883	0.061
BG01_02	0.571*	0.078	0.674	0.090
BG01_06	0.542*	0.079	0.706	0.085
BG01_07	0.590*	0.079	0.652	0.094
**GS (Professionalism towards society)**				
GS01_01	0.316*	0.096	0.900	0.060
GS01_03	0.807*	0.084	0.350	0.135
GS01_04	0.655*	0.078	0.571	0.102
	Coef.	OIM SE		
cov(ε.SE02_03*ε.SE02_28)	0.387*	0.073		
cov(ε.SE02_07*ε.SE02_20)	0.383*	0.072		
cov(ε.BG01_01*ε.BG01_02)	0.502*	0.071		
cov(ε.BG01_06*ε.BG01_07)	0.511*	0.075		
cov(SE*PA)	0.487*	0.091		
cov(SE*BG)	0.810*	0.099		
cov(SE*GS)	0.402*	0.108		
cov(PA*BG)	0.747*	0.108		
cov(PA*GS)	0.430*	0.103		
cov(BG*GS)	0.628*	0.124		
Notes: SEM output of the confirmatory factor analysis with 30 *MediProf* items and the four questionnaire dimensions as latent, standardised (=1) factors. Items were excluded from the model if item-total correlation <0.3 and/or item difficulty >0.8 (see **Tables A3.1–A3.4**). Full-information maximum likelihood (FIML) estimation was applied. Covariances between error terms were added based on modification indices (Lagrange multiplier tests) to improve model fit. SE: standard error. N = 155. LR test (model vs. saturated): χ^2 (367) = 647.46; Prob > χ^2: $p < 0.001$. *: $p < 0.05$.				

Table A3.6: Confirmatory factor analysis (30 *MediProf* items)
